# Supplementary material for: Hispolon Cyclodextrin Complexes and Their Inclusion in Liposomes for Enhanced Delivery in Melanoma Cell Lines
Source: Int J Mol Sci. 2022 Nov 21;23(22):14487. doi: 10.3390/ijms232214487 (PMC9695989; doi:10.3390/ijms232214487)

Supplementary Information

Figure S1. Calibration curve of Hispolon standard solutions ranging from (2 µg/ml-100 µg/ml).

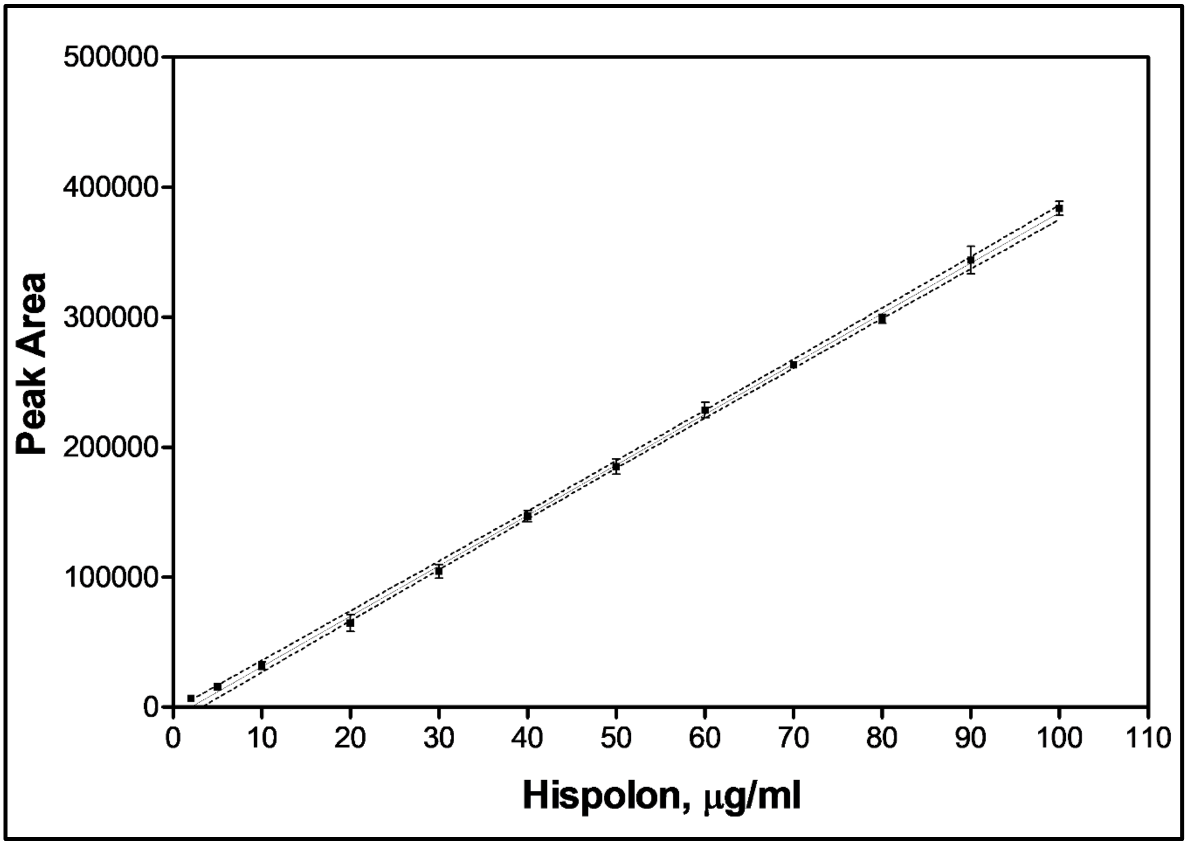

Figure S2. Representative Chromatogram of a Hispolon Standard Solution(100 µg/ml).

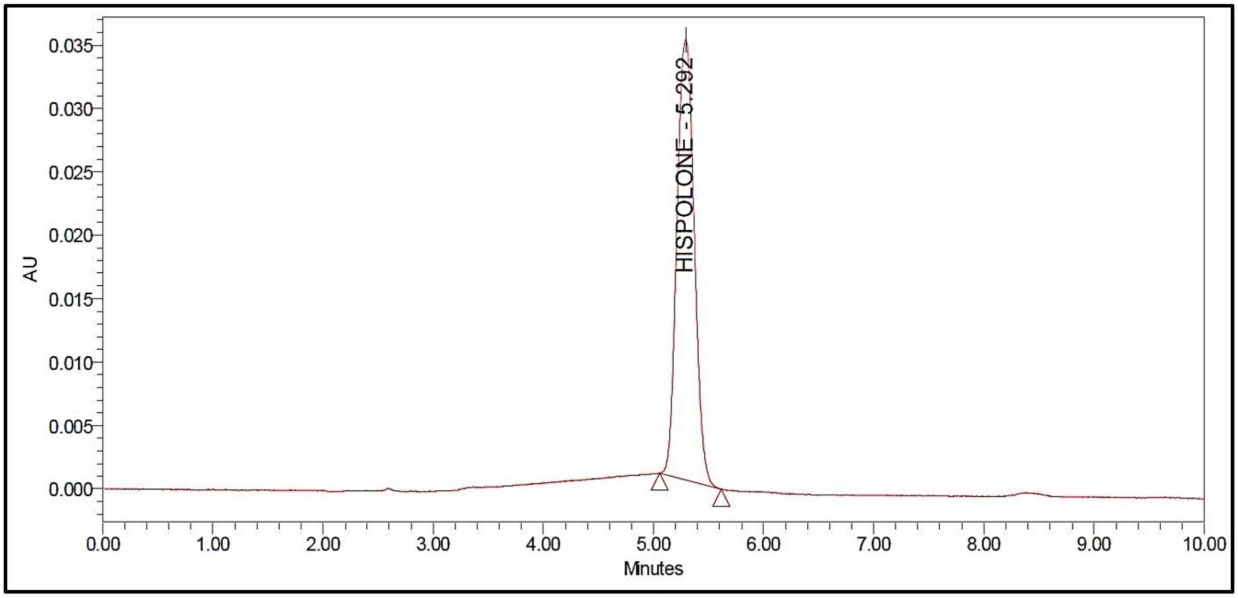

Figure S3. Job's continuous variation plot of Hispolon-SBEβCD (HSC) complex. Data represented as mean ± SEM (n=3).

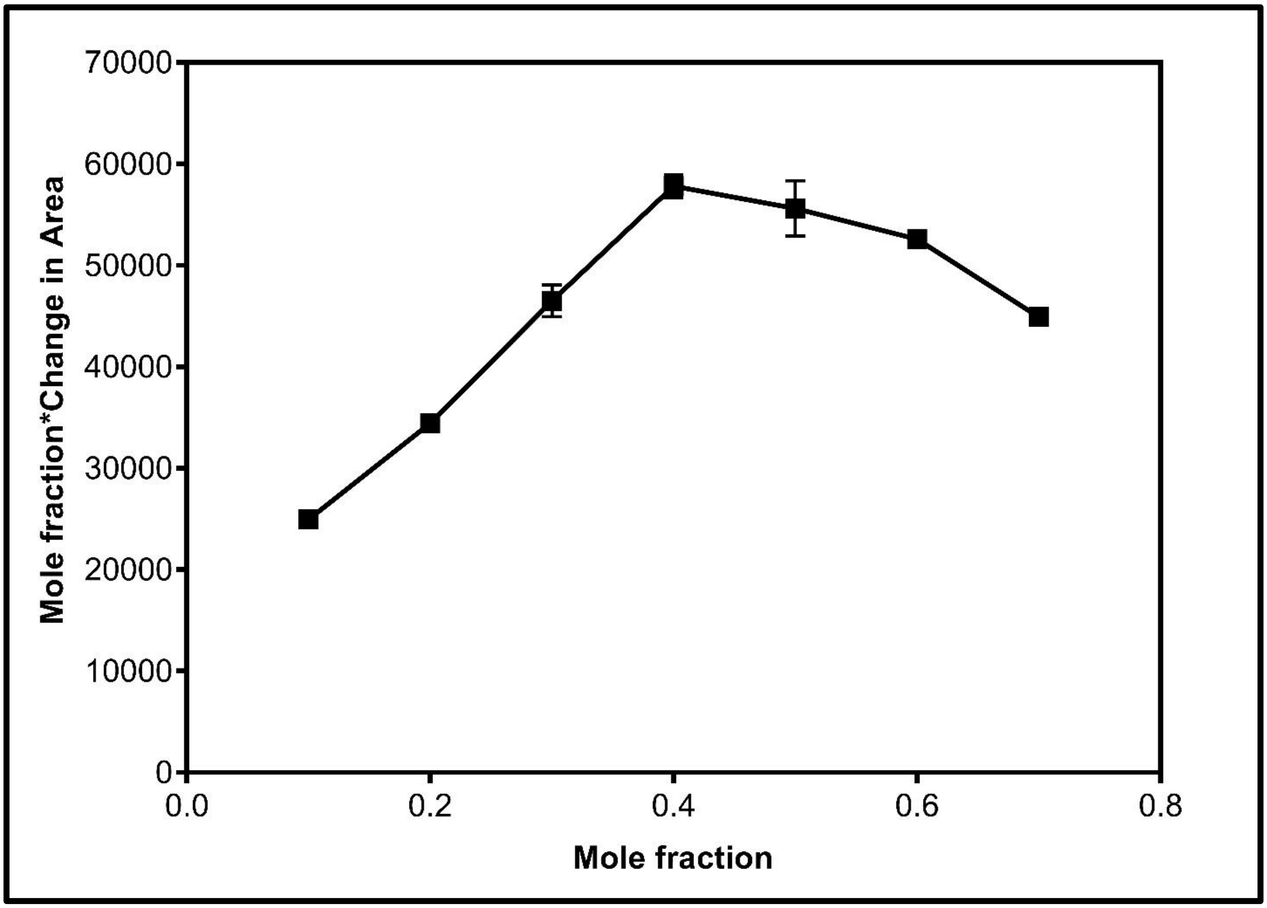

Supplement: Supplementary file 1 [file ijms-23-14487-s001.zip › ijms-1991779-supplementary.pdf]
